# Associations with kidney transplant survival and eGFR decline in children and young adults in the United Kingdom: a retrospective cohort study

**DOI:** 10.1186/s12882-020-02156-2

**Published:** 2020-11-18

**Authors:** Alexander J. Hamilton, Lucy A. Plumb, Anna Casula, Manish D. Sinha

**Affiliations:** 1 Population Health Sciences, University of Bristol, G.04, Canynge Hall, 39 Whatley Road, Bristol, BS8 2PS UK; 2 UK Renal Registry, Bristol, UK; 3 Evelina London Children’s Hospital, London, UK; 4 Kings College London, London, UK

**Keywords:** Kidney transplant, Child, Adolescent, Young adult, Graft survival, eGFR

## Abstract

**Background:**

Although young adulthood is associated with transplant loss, many studies do not examine eGFR decline. We aimed to establish clinical risk factors to identify where early intervention might prevent subsequent adverse transplant outcomes.

**Methods:**

Retrospective cohort study using UK Renal Registry and UK Transplant Registry data, including patients aged < 30 years transplanted 1998–2014. Associations with death-censored graft failure were investigated with multivariable Cox proportional hazards. Multivariable linear regression was used to establish associations with eGFR slope gradients calculated over the last 5 years of observation per individual.

**Results:**

The cohort (*n* = 5121, of whom *n* = 371 received another transplant) was 61% male, 80% White and 36% had structural disease. Live donation occurred in 48%. There were 1371 graft failures and 145 deaths with a functioning graft over a 39,541-year risk period. Median follow-up was 7 years. Fifteen-year graft survival was 60.2% (95% CI 58.1, 62.3).

Risk associations observed in both graft loss and eGFR decline analyses included female sex, glomerular diseases, Black ethnicity and young adulthood (15–19-year and 20–24-year age groups, compared to 25–29 years). A higher initial eGFR was associated with less risk of graft loss but faster eGFR decline. For each additional 10 mL/min/1.73m^2^ initial eGFR, the hazard ratio for graft loss was 0.82 (95% CI 0.79, 0.86), *p* < 0.0001. However, compared to < 60 mL/min/1.73m^2^, higher initial eGFR was associated with faster eGFR decline (> 90 mL/min/1.73m^2^; − 3.55 mL/min/1.73m^2^/year (95% CI -4.37, − 2.72), *p* < 0.0001).

**Conclusions:**

In conclusion, young adulthood is a key risk factor for transplant loss and eGFR decline for UK children and young adults. This study has an extended follow-up period and confirms common risk associations for graft loss and eGFR decline, including female sex, Black ethnicity and glomerular diseases. A higher initial eGFR was associated with less risk of graft loss but faster rate of eGFR decline. Identification of children at risk of faster rate of eGFR decline may enable early intervention to prolong graft survival.

**Supplementary Information:**

The online version contains supplementary material available at 10.1186/s12882-020-02156-2.

## Background

Kidney transplantation is the optimal treatment for end stage kidney disease (ESKD) in children and young adults as it provides them the best opportunity for normal growth and development, a better quality of life and places fewer restrictions on daily activities [1]. For health service providers, transplantation is also the most cost-effective kidney replacement therapy (KRT) modality [2].

Young adulthood though remains a distinct period for risk of graft loss. A large registry study analysing the effect of age identified graft failure rates were highest at 19 years and that those aged 17–24 years were at the highest risk of graft failure [3], with similar findings from other studies not including patients from the UK [4–7]. This may be because both adolescence and young adulthood are associated with increased risk-taking behaviour, poor medication compliance and changes to care as patients transfer to adult services [3, 5]. Furthermore, brain development continues during young adulthood [8], which may impact reasoning and decision-making. Thus, following periods of stable graft function, typically, this may be followed by period of worsening graft function and potential sudden graft failure.

There are few studies including data reporting UK graft survival for children and young adults. These data are though limited for their generalisability as one reported outcomes for transplants between 1973 and 2000 [9] and another described outcomes for deceased donor transplant recipients alone [10]. Two further reports have data on short term outcomes limited to 3–5 years with a specific focus on HLA mismatching: non-inferiority of poorly matched living donor transplants compared to well-matched deceased transplants suggested by one [11], with opposing evidence for this in the larger Collaborative Transplant Study dataset [12]. A recent study reported graft loss outcomes for UK young adults using Hospital Episode Statistics and Office for National Statistics data and showed an increased risk of graft loss relative to other age groups for those aged 14–23 years with 10-year follow up [13].

Although it is unlikely that outcomes during adolescence and young adulthood will differ for UK individuals, to our knowledge no studies have evaluated the role of initial transplant function or examine risk factors for declining function. Importantly, studies have been limited in their focus and duration of follow up, with the maximum period of follow up limited to 10 years. This follow-up period is relevant as between 2000 and 2011, UK children received live and donation after brain death kidney transplants at a mean age of 11 ± 5 years [11]. The median age at transfer to adult services in the UK is 18 years [14]. Therefore following 10 years of follow up a large proportion would not yet have transferred and many would be under the age of 24 years, reported as the upper limit of the high-risk age window [3].

In this study, we aimed to use explanatory regression models to establish clinical characteristics for early identification of those at risk of declining transplant function and failure using data collected across two major registries that include all children and young adults with follow up data extending beyond childhood and > 10 years where available. This would allow for the development of targeted interventions intending to preserve transplant function and prevent graft loss. We report graft survival for UK children and young adults and to explore the importance of clinical donor and recipient variables not only for graft loss but also rate of decline, among grafts of varying vintage. To our knowledge, factors associated with graft function decline in children and young adults have not previously been evaluated. Clinical identification of eGFR decline is time-critical for this group and intervention often requires a biopsychosocial approach.

## Methods

We undertook a retrospective cohort study using linked UK Renal Registry (UKRR) and NHS Blood and Transplant (NHSBT) data, including children and young adults aged < 30 years who underwent kidney transplantation between 1998 and 2014. As UKRR and NHSBT datasets differ, their combination provided comprehensive clinic-demographic data with greater completeness and reliability.

### Clinical data from the UKRR and NHSBT

The UKRR collects data on all patients receiving KRT from UK adult and pediatric kidney units [15, 16]. It has been granted a section 251 exemption by the Health Research Authority, allowing the registration of identifiable patient information from kidney units without first asking individual patient consent. We defined exposures and covariates in a research protocol and applied for data in October 2017, receiving approval in February 2018. The data was received December 2018–March 2019. The study included all patients aged < 30 years who received a kidney transplant from 01/01/1998 to 31/12/2014. These dates were chosen to reflect full coverage UK kidney units and to ensure at least 1 year of individual follow-up time. Biochemical data was extracted up to the final quarter of 2016. Initial eGFR was calculated from the first biochemical data recorded following transplantation; returns are annual for paediatrics and quarterly for adults. Patients may have previously received a transplant prior to the study period.

NHSBT is a special health authority supporting UK organ transplantation and collects data on transplant outcomes for the UK Transplant Registry (UKTR). Informed consent for data collection is obtained from recipients and donor next of kin. We applied for data in November 2017. The data was extracted in October 2018 and included follow-up to this time point, hence was the most recent and used in analyses. NHBT describes human leucocyte antigen (HLA) mismatches according to the UK 2006 National Kidney Allocation scheme, which emphasises DR-locus mismatches to stratify risk [17].

### Statistical analyses

#### Graft loss

We modelled death-censored graft failure over death as a competing risk, as this is felt to be more appropriate for an aetiological research question [18], aiming to identify clinical risk factors. Univariate analyses included Kaplan-Meier survival curves and log-rank tests. We used a conditional risk-set model for multiple failure data [19]. This measures time to event from the time of the previous event, with follow-up time reset to zero after each failure event where patients were re-transplanted. So, when retransplanted, the same individual appears twice in the data, as follows:

**Table:** 

ID	*Graft number*	*Entry time*	*Exit time*	*Censored*
1	1	0	2.17	1
1	2	0	13.9	0

By using a multivariable Cox proportional hazards model, we explored the association of evidence-based variables as well as initial eGFR post-transplant on the risk of death-censored graft failure [hazard ratio (HR) (95% confidence interval (CI)), *p*-value]. We analysed age group as time-varying and used stratification by graft number, clustering at the participant level (to account for some participants appearing more than once in the dataset) and Efron’s method for handling ties [20]. We tested for the assumption of proportionality using visual plots, Schoenfeld residuals and testing for a log–time interaction. As some indication of non-proportionality was observed for glomerular diseases versus other kidney diseases, we also performed piecewise Cox regression to confirm the results.

#### eGFR trend

We chose to explore eGFR decline assuming linearity given that this is a widely used and well-established method [21–23]. We calculated estimated glomerular filtration rate (eGFR) using the Schwartz formula [24] if aged < 18 years and the 4-variable Modification of Diet in Renal Disease formula [25] otherwise. Although it has been suggested to average pediatric and adult serum Creatinine-based eGFR equations for those aged 18–26 years [26], our approach reflects current UK clinical practice. Where height was missing, the previous entry was substituted. Where ethnicity was missing, white ethnicity was substituted. For participants with ≥3 eGFR values outside the first 6 months post-transplant, individual linear regressions (least squares) of eGFR against time were performed using the most recent 5 years of data per participant. For example, for someone transplanted in 2005 with a functioning graft throughout the study period, we used data from 2012 to 2016. Analysis of patient-level eGFR slopes suggested that linear modelling provided a reasonably accurate fit for the time-period of kidney function explored. Using the most recent 5 years of observation resulted in greater linearity than using all available data and less combined use of eGFR equations in the same individual. Since eGFR was calculated to create a regression line, the equation used was of less importance as regression line gradients would be similar, and the regression line would smooth any missing eGFR values. For quarterly data, the data collection quarter mid-point was used.

We used backward elimination and examined explanatory variables in univariate models to establish associations with eGFR slope gradients. We combined associated variables (*p* < 0.05) in a multivariable linear (least squares) regression model and removed explanatory variables from the final model if there was no statistical association when co-adjusted, having checked that this did not affect the regression coefficients of the remaining variables. We checked for assumptions of linearity between continuous variables and the outcome variable, evidence of heteroscedasticity and normally distributed residuals with a mean of zero. Where non-linear, continuous variables were categorised into clinically relevant groups. We tested for potential pre-specified interactions: 1) sex and primary kidney disease, 2) initial eGFR and sex, 3) ethnicity and primary kidney disease, 4) initial eGFR and ethnicity (as ethnicity contributes to eGFR equations). Data are reported appropriate to their distribution. We used Stata v.15 for our analyses.

#### First eGFR post-transplant

Data returns to the UKRR are annual for paediatric patients and quarterly for adult patients. The first serum creatinine result reported to the UKRR post-transplant for paediatrics is at 3 months (alongside a height measurement) and within the first 3 months for adults. These data were used to derive the first eGFR measurement in the first 3 months following transplantation, thus reflecting early steady state.

## Results

Completeness was high for most items (Table 1). Deaths were discordant between UKRR and UKTR datasets. All patients with a coded UKTR death and no UKRR death (*n* = 32) died after the final date in the UKRR data extraction. Almost all the patients with a coded UKRR death and no UKTR death (*n* = 45) died after the follow-up time for graft and patient outcomes in the UKTR data. The remaining patient (*n* = 1) had biochemical data submitted after the death date and is a likely error. As the UKTR outcome data was used this did not affect the graft loss analysis.

**Table 1:** Patient and transplant characteristics

Variable	Total n	n	%
**Male sex**	5121	3107	60.7
**Ethnic group**			
White	4918	3955	80.4
Asian		579	11.8
Black		215	4.4
Mixed/other		169	3.4
**Primary kidney disease group**^**a**^			
Glomerular disease	5065	1467	29.0
Systemic diseases affecting the kidney		304	6.0
Familial/hereditary nephropathies		593	11.7
Tubulointerstitial disease		1808	35.7
Miscellaneous kidney disorders		893	17.6
**Late presentation**^**b**^	3366	867	25.8
**Age when first seen (years) (median, IQR)**	3389	13.1	2.7, 20.0
**Age at KRT start (years) (median, IQR)**	5085	19.1	12.9, 24.1
**Years to KRT start from first nephrology review (median, IQR)**	3370	2.0	0.2. 5.4
**Tertiles of year started KRT (median, IQR)**	5085	2006	2001, 2009
1983–2003	5085	1936	38.1
2004–2008		1607	31.6
2009–2014		1542	30.3
**Start modality**			
Haemodialysis	5102	2011	39.4
Peritoneal dialysis		1839	36.0
Transplant		1252	24.5
** Age at transfer (years) (median, IQR)**	1455	18	17.4, 18.5
**Died**	5121	260	5.1
** Age at death (years) (median, IQR)**	260	27.6	20.3, 32.3
**Transplanted during study period**			
Graft 1	4750	4377	85.5
Graft 2		371	7.2
Graft 3		2	0.04
** Re-transplanted during study period**			
Graft 2	371	369	7.2
Graft 3		2	0.04
**Time from listing to transplant (years) (median, IQR)**	4416	0.8	0.3, 1.8
**Year of transplant**			
1998–2003	5121	1291	25.2
2004–2009		1930	37.7
2010–2014		1900	37.1
**Donor type**			
Live donation	5121	2462	48.1
Donation after brainstem death		2356	46.0
Donation after circulatory death		303	5.9
**Donor age (years) (median, IQR)**	5115	41	27, 49
**Cold ischaemic time (hours) (median, IQR)**	4651	9.3	2.8, 16.3
**Calculated reaction frequency (%) (median, IQR)**^**c**^	5118	0	0, 12
**HLA mismatch group**^**d**^			
0 mismatches	5120	544	10.6
[0 HLA-DR and 0/1 HLA-B] mismatches		2016	39.4
[0 HLA-DR and 2 HLA-B] or [1 HLA-DR and 0/1 HLA-B] mismatches		2272	44.4
[1 HLA-DR and 2 HLA-B] or [2 HLA-DR] mismatches		288	5.6
**First eGFR (mL/min/1.73m**^**2**^**) (mean, SD)**^**e**^	4882	62	23
**Rate of eGFR change (mL/min/1.73m**^**2**^**/year) (mean, SD)**	4487	−3.14	7.69
**Graft failure**	5111	1376	26.9
Follow up time (years) (median, IQR)	5111	7.0	4.0, 10.9
Time to event (years) (median, IQR)	1376	4.2	1.5, 7.6
**Patient death**	4750	238	5.0
Follow up time (years) (median, IQR)	4750	8.8	5.1, 13.0
Time to event (years) (median, IQR)	238	6.6	3.0, 10.6
**Age at event (mean, SD)**	5111	27.5	8.7

*IQR* interquartile range, *KRT* kidney replacement therapy, *SD* standard deviation, *HLA* human leucocyte antigen, *eGFR* estimated glomerular filtration rate

Ages are based on dates of birth rounded to mid-month

^a^Primary kidney disease was using a 2012 European coding system [27]. The pediatric diagnosis was used where discordant between pediatric and adult databases [28]

^b^Late presentation defined as ≤90 days from first nephrology review to RRT start

^c^Calculated reaction frequency is defined as the percentage of ABO-identical patients within the donor pool that are HLA incompatible with an individual patient and is dependent on blood group and antibodies [29]

^d^HLA mismatch groups were derived from the UK 2006 National Kidney Allocation scheme [17]. The HLA-A:B:DR mismatches included in each group are as follows:

[0 HLA-DR and 0/1 HLA-B] - 1:0:0, 0:1:0, 1:1:0, 2:0:0, 2:1:0

[0 HLA-DR and 2 HLA-B] or [1 HLA-DR and 0/1 HLA-B]

0:2:0, 1:2:0, 2:2:0, 0:0:1, 1:0:1, 2:0:1, 0:1:1, 1:1:1, 2:1:1

[1 HLA-DR and 2 HLA-B] or [2 HLA-DR]

0:2:1, 1:2:1, 2:2:1, 0:0:2, 1:0:2, 2:0:2, 0:1:2, 1:1:2, 2:1:2, 0:2:2, 1:2:2, 2:2:2.

^e^eGFR post-transplant calculated from the first biochemical data recorded by the UK Renal Registry following transplantation. Returns are annual for paediatrics and quarterly for adults

Clinical characteristics are displayed in Table 1, with additional detail in Supplemental Table 1. The cohort (*n* = 5121) mostly included one graft during the study period (*n* = 4750, this was predominantly the patient’s first ever transplant (*n* = 4377) but it may have been their second (*n* = 371) or third (*n* = 2) kidney). Seven percent (*n =* 371) received an additional transplant during the study period, primarily the patient’s second transplant (*n* = 369) with third transplants being uncommon (*n =* 2). The median age at the patient’s first transplant was 21 years (interquartile range (IQR) 14, 26). The cohort was 61% male, 80% white and 36% had ESKD due to structural kidney disorders. A quarter started KRT within 90 days of their first nephrology review. Pre-emptive transplantation occurred in 25%. Median kidney transplant waitlist time was 0.8 years (IQR 0.3, 1.8). Participants lived a median of 26.2 km (IQR 10.7, 54.8) from their transplant centre. Live donation occurred in 48%. Median cold ischaemic time was 9.3 h (IQR 2.8, 16.3). HLA matching was considered ‘good’ (either no mismatches or 0 HLA-DR and 0/1 HLA-B mismatch) in 50% [17]. Mean first eGFR was 62 mL/min/1.73m^2^ (see Table 1 footnote), with a mean linear change of − 3 mL/min/1.73m^2^/year over the most recent five years of follow-up.

**Fig. 1:**
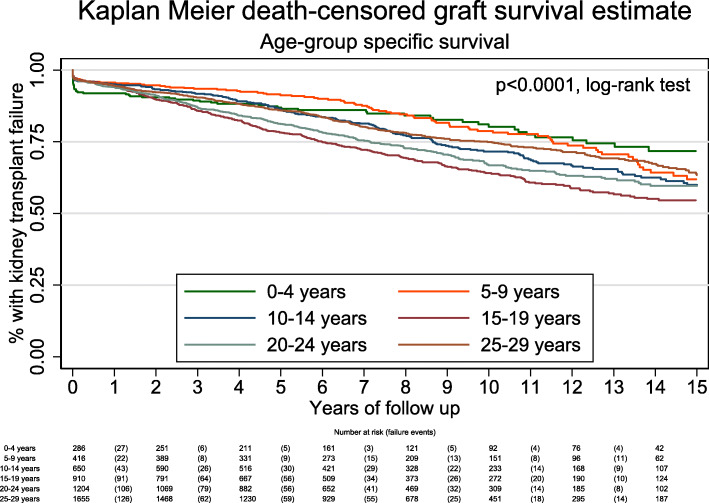
The youngest age group had the highest initial kidney transplant failure rate but the best long-term transplant survival. Young adults (aged 15–24 years) had the poorest long-term transplant survival

There were 1371 graft failures and 145 deaths with a functioning graft over a 39,541-year risk period. Median follow-up was 7 years. Graft survival was as follows: 1-year 94.4% (95% CI 93.7, 95.0), 5-year 84.0% (95% CI 82.9, 85.0), 10-year 71.1% (95% CI 69.6, 72.5), 15-year 60.2% (95% CI 58.1, 62.3) and 20-year 51.2% (95% CI 47.6, 54.7%). Graft survival varied by age at transplant and changed over time (Fig. 1); although those transplanted aged 0–4 years had the least initial graft survival, by 10 years they had the highest graft survival. Those transplanted aged 15–19 years had the fastest rate of transplant failure. At 15 years, those transplanted aged 0–4 years had a survivor function of 71.7% (95% CI 63.4, 78.5) compared to 54.4% (95% CI 49.9, 58.7) for those transplanted aged 15–19 years, *p* < 0.0001.

**Fig. 2:**
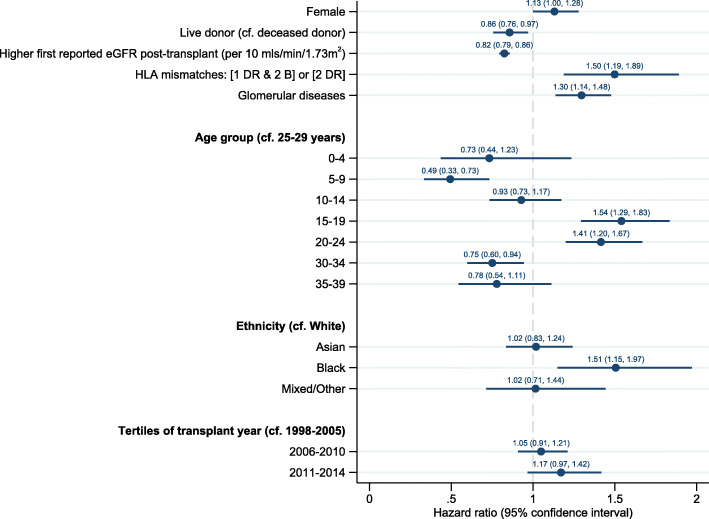
An increased hazard ratio of death-censored kidney transplant failure was associated with female sex, deceased kidney donation, lower initial kidney transplant function, more HLA mismatches, young adulthood (age group 15–24), Black ethnicity and glomerular diseases. *N* = 4392. eGFR, estimated glomerular filtration rate; HLA, human leucocyte antigen

Associations with graft loss from the multivariable Cox proportional hazards model are shown in Table 2 and visually in Fig. 2. Graft loss varied over the age spectrum; compared to 25–29 years, being aged 5–9 years was a protective association (HR 0.49 (95% CI 0.33, 0.73), *p* < 0.0001), whereas young adulthood was a risk association (15–19 years, HR 1.54 (95% CI 1.29, 1.83), *p <* 0.0001; 20–24 years, HR 1.41 (95% CI 1.20, 1.67), *p <* 0.0001). Older age was associated with reduced risk of graft loss (30–34 years, HR 0.75 (95% CI 0.60, 0.94), *p* = 0.01). Other protective associations were having a live donor (HR 0.86 (95% CI 0.76, 0.97), *p =* 0.01) and higher initial eGFR (per 10 mL/min/1.73m^2^) (HR 0.82 (95% CI 0.79, 0.86), *p* < 0.0001). Risk associations included female sex (HR 1.13 (95% CI 1.00, 1.28), *p* = 0.049, adverse HLA mismatches (HR 1.50 (95% CI 1.19, 1.89), *p* = 0.001), Black ethnicity (HR 1.51 (95% CI 1.15, 1.97), *p* = 0.003), and glomerular diseases (HR 1.30 (95% CI 1.14, 1.48), *p <* 0.0001). We found no statistical relationship with time period, calculated reaction frequency, socio-economic status or pre-emptive transplantation in multivariable analyses. Univariable hazard ratios are shown in Supplemental Table 2. A piecewise model split at one year is shown in Supplemental Table 3.

**Table 2:** Multivariable Cox proportional hazards examining associations with death-censored kidney transplant failure in UK patients transplanted under 30 years of age

Variable	Hazard Ratio	95% confidence interval		***p***-value
		Lower	Upper	
**Female sex**	**1.13**	1.00	1.28	0.049
**Live donor (cf. deceased donor)**	**0.86**	0.76	0.97	0.01
**Human Leucocyte Antigen mismatches**^**a**^ [One DR & two B locus OR two DR locus mismatches]	**1.50**	1.19	1.89	0.001
**Higher first reported eGFR post-transplant**^**b**^ (per 10 mL/min/1.73 m^2^)	**0.82**	0.79	0.86	< 0.0001
**Glomerular diseases**^**c, d**^	**1.30**	1.14	1.48	< 0.0001
**Age group (cf. 25–29 years)**^**e**^				
2–4	**0.73**	0.44	1.23	0.2
5–9	**0.49**	0.33	0.73	< 0.0001
10–14	**0.93**	0.73	1.17	0.5
15–19	**1.54**	1.29	1.83	< 0.0001
20–24	**1.41**	1.20	1.67	< 0.0001
30–34	**0.75**	0.60	0.94	0.01
35–39	**0.78**	0.54	1.11	0.2
40–44	**0.82**	0.39	1.70	0.6
**Ethnicity (compared to White)**				
Asian	**1.02**	0.83	1.24	0.9
Black	**1.51**	1.15	1.97	0.003
Mixed/Other	**1.02**	0.71	1.44	0.9
**Year of transplant (cf. 1998–2005)**				
2006–2010	**1.05**	0.91	1.21	0.5
2011–2014	**1.17**	0.97	1.42	0.1

Stratified by transplant number in the study period. Standard error adjusted for 4392 clusters

Live donation by ethnic group was as follows: White, 49%; Asian, 32%, Black, 37%; Mixed/Other, 48%

^a^HLA mismatch groups were derived from the UK 2006 National Kidney Allocation scheme [17]

^b^eGFR post-transplant calculated from the first biochemical data recorded by the UK Renal Registry following transplantation. Returns are annual for paediatrics and quarterly for adults

^c^Primary kidney disease was using a 2012 European coding system [27]. The pediatric diagnosis was used where discordant between pediatric and adult databases [28]

^d^There was non-proportionality over time between those with and without glomerular diseases. Piecewise Cox regression analyses split at 1 year showed similar effects for glomerular diseases [≤1 year HR 2.05 (1.34, 3.14), *p =* 0.001; > 1 year HR 1.24 (1.08, 1.43), *p* = 0.002) and no effect on other HRs; this model presents the overall HR for the entire follow-up period

^e^The 45–49-year age group is suppressed due to small numbers (*n* = 8)

**Fig. 3:**
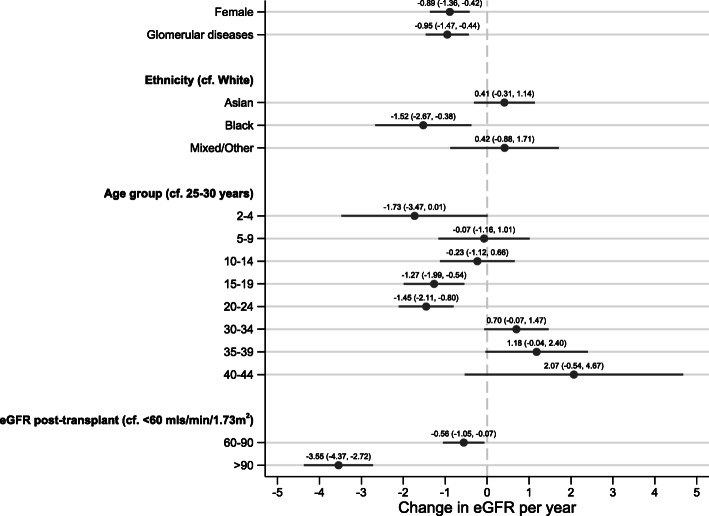
eGFR decline was associated with female sex, glomerular diseases, Black ethnicity, young adulthood (age group 15–24) and higher initial kidney transplant function. *N* = 4284. The regression coefficient of the model intercept was − 1.54 (95% CI, − 2.08 to − 1.00; *p <* 0.0001). The model R^2^ was 0.04. eGFR, estimated glomerular filtration rate

Associations with eGFR decline are shown in Table 3 and visually in Fig. 3. Regression coefficients represent rate of annual eGFR change (mL/min/1.73 m^2^/year). Risk associations for eGFR decline included female sex (− 0.89 mL/min/1.73m^2^/year (95% CI -1.36, − 0.42), *p* < 0.0001), glomerular diseases (− 0.95 mL/min/1.73 m^2^/year (95% CI -1.47, − 0.44), *p <* 0.0001), young adulthood, Black ethnicity and higher initial transplant function. Compared to being aged 25–29 years, being aged 15–24 years was associated with eGFR decline (15–19 years, − 1.27 mL/min/1.73 m^2^/year (95% CI -1.99, − 0.54), *p* = 0.001; 20–24 years, − 1.45 mL/min/1.73 m^2^/year (95% CI -2.11, − 0.80), *p* < 0.0001). Compared to White, Black ethnicity was associated with eGFR decline (− 1.52 mL/min/1.73 m^2^/year (95% CI -2.67, − 0.38), *p* = 0.009). Compared to an initial eGFR of < 60 mL/min/1.73m^2^, higher initial eGFR was associated with eGFR decline (60–90 mL/min/1.73m^2^, − 0.56 mL/min/1.73 m^2^/year (95% CI -1.05, − 0.07), *p* = 0.03); > 90 mL/min/1.73m^2^, − 3.55 mL/min/1.73 m^2^/year (95% CI -4.37, − 2.72), *p <* 0.0001). A significant interaction (*p <* 0.0001) was found between ethnicity and initial eGFR, with stratum-specific exposure effects detailed in Table 3. The most clinically relevant was for Black ethnicity and initial eGFR > 90 mL/min/1.73 m^2^, associated with an eGFR decline of − 9.00 mL/min/1.73 m^2^/year (95% CI -12.6, − 5.44); *p* < 0.0001. Conversely, there was no statistical difference in rate of eGFR decline for other ethnic groups across initial eGFR groups. There were no interactions between sex and primary kidney disease, sex and initial eGFR or ethnicity and primary kidney disease. Univariable coefficients are shown in Supplemental Table 4.

**Table 3:** Multivariable linear regression examining associations with eGFR decline in UK patients transplanted under 30 years of age

Variable	Coefficient	95% confidence interval		***p***-value
		Lower	Upper	
**Female sex**	**−0.89**	−1.36	−0.42	< 0.0001
**Glomerular diseases**^**a**^	**−0.95**	− 1.47	− 0.44	< 0.0001
**Ethnicity (cf. White)**				
Asian	**0.41**	−0.31	1.14	0.3
Black	**−1.52**	−2.67	−0.38	0.009
Mixed/Other	**0.42**	−0.88	1.71	0.5
**First reported eGFR post-transplant**^**b**^ **(cf. < 60 mL/min/1.73m**^**2**^**)**				
60–90	**−0.56**	−1.05	−0.07	0.03
> 90	**−3.55**	−4.37	−2.72	< 0.0001
**Age group**^**c**^ **(cf. 25–29 years)**				
2–4	**−1.73**	−3.47	0.01	0.05
5–9	**−0.07**	−1.16	1.01	0.9
10–14	**−0.23**	−1.12	0.66	0.6
15–19	**−1.27**	− 1.99	−0.54	0.001
20–24	**−1.45**	−2.11	−0.80	< 0.0001
30–34	**0.70**	−0.07	1.47	0.08
35–39	**1.18**	−0.04	2.40	0.06
40–44	**2.07**	−0.54	4.67	0.1

^a^Primary kidney disease was using a 2012 European coding system [52]. The pediatric diagnosis was used where discordant between pediatric and adult databases [28]

^b^eGFR post-transplant calculated from the first biochemical data recorded by the UK Renal Registry following transplantation. Returns are annual for paediatrics and quarterly for adults. There was evidence of non-linearity between initial eGFR and eGFR decline and therefore data are presented as categorical

^c^Age at start of eGFR slope analysis. Age range grouped into 5-year bands, however there were no transplants under the age of 2 years

The regression coefficient of the model intercept was −1.54 (95% CI, − 2.08 to − 1.00; *p* < 0.0001)

There were no interactions between: sex and primary kidney disease, sex and initial eGFR or primary kidney disease and ethnicity. There was a significant interaction (likelihood ratio test *p* < 0.0001 between full model and model fitting the interaction term) between initial eGFR and ethnicity. The stratum-specific exposure effects of initial eGFR and ethnicity (compared with White, eGFR < 60 mL/min/1.73 m^2^) are as follows:

Asian, eGFR 60–90 0.11 (95% CI -1.45, 1.66), *p* = 0.9

Asian, eGFR > 90 -0.49 (95% CI -2.73, 1.76), *p* = 0.7

Black, eGFR 60–90 0.30 (95% CI -2.17, 2.77), *p* = 0.8

Black, eGFR > 90 -9.00 (95% CI -12.6, − 5.44), *p <* 0.0001

Mixed/Other, eGFR 60–90 -0.34 (95% CI -3.08, 2.40), *p =* 0.8

Mixed/Other, eGFR > 90 -2.25 (95% CI -6.55, 2.05), *p* = 0.3

## Discussion

This study reports long-term graft survival for UK children and young adults and evaluates associations with both transplant failure and eGFR decline. This study provides an extended follow-up period relative to the established literature using high-quality linked datasets; this was necessary to develop a complete dataset of biochemical data and transplant outcomes and enabled comprehensive analysis of relevant clinical variables. In addition to being a risk factor for graft loss, it highlights that young adulthood is associated with eGFR decline in individuals transplanted before the age of 30 years. Risk associations for both outcomes included female sex, young adulthood (15–24 years), glomerular diseases and Black ethnicity. Initial transplant function was bidirectionally associated with both outcomes, with better initial function being associated with less risk of graft loss but a faster rate of eGFR decline. There are scarce data on eGFR decline among children and young adults, yet graft health is considered a core outcome for research [30]. Whilst it might be assumed that risk factors may be similar for eGFR decline and graft loss, this can now be confirmed.

This study confirms the association between young adulthood and graft loss for UK patients. Compared to 25–29 years, being aged 15–24 years is associated with faster eGFR decline, but children reaching this age have a higher risk of graft loss, irrespective of time from transplant. Most UK patients transfer to adult centres at 18 years, suggesting age-related risks are not solely about transition of care and interventions may be required across pediatric and adult services. Young adults on KRT need psychosocial support. Compared to the general population, they have impaired relationships, independence, employment, quality of life, mental wellbeing and psychological health [31, 32]. Low medication adherence is common, affecting 43% [33]. Young adults live with uncertainty, thwarted ambitions, body image issues and social isolation [34]. Healthcare services for young people vary internationally, with different UK models proposed [35, 36]. However, UK psychologists and social worker provision falls short of workforce recommendations [37, 38].

High rates of graft loss for young patients have been reported, but with limited follow-up [3]. Five-year European graft survival for children has been reported at 88% [39]. Our extended follow-up period enabled observation of better 10-year graft survival for children aged 0–4 years relative to other age groups, despite an early increased risk of failure. Younger patients are more likely to have a live donor (55% of the 0–4 year age group, compared to 48% of the 25–29 year age group, *p* = 0.04) or a deceased transplant from a younger donor (median donor age in the 0–4 year age group was 23 years (IQR 23, 41) compared to 39 years (IQR 24, 50) in the 25–29 year age group, *p* < 0.0001). These factors are associated with a lower risk of graft loss [5, 40, 41]. As would be expected, young children aged 5–9 were low risk for death-censored kidney transplant loss. In these young children medications will be administered by parents and compliance less likely to be of concern. Young age may confer an immunological advantage, with a relatively naïve adaptive immunity more capable of accommodating the foreign graft [42].

Our findings confirm the association of female sex, Black ethnicity and glomerular diseases with graft loss [3, 5]. Black ethnicity is associated with poor graft outcomes in UK adults, although this may be mediated by reduced access to live donors and HLA mismatching, perhaps due to inherent biological ethnic differences [43]. Our study categorised HLA group emphasising HLA-DR locus mismatches to stratify risk, contrasting with non-UK studies which tend to report total mismatches. Using this classification, we found that adverse mismatches were associated with graft loss but not with eGFR decline, possibly due to the rarity of adverse mismatches (6%). Whilst shown to be associated with graft loss in adult populations [44, 45], few studies report the impact of initial transplant function in children and young adults. One study reported graft survival benefits with an eGFR ≥30 mL/min/1.73 m^2^ at 6 months post-transplant, among other factors [46]. Higher initial eGFR appears protective for graft loss within this population, independent of HLA mismatching and living donation. This indicates benefit from good-quality organs and the need to minimise established factors (e.g. cold ischemic time) known to negatively impact graft survival.

In the UK, a return to dialysis is estimated to cost an additional £10,000 per year, however failing grafts may lead to the need for increased appointments and psychological support, ongoing immunosuppression, management of anaemia and metabolic bone disease, dialysis planning and transplant nephrectomy [47, 48], as well as frailty, incurring higher costs. In this study, we also sought to determine factors predictive of eGFR decline. Contrary to graft loss, which is a late and less frequent outcome, rate of graft decline is a ‘real-time’ measure often used by clinicians and families to gauge graft health post-transplant. Although the risk factors identified are inherently non-modifiable, they can 1) help clinicians to identify patients who may benefit from enhanced care and 2) help patients to understand and manage expectations of their transplant function. Future work could include the derivation and validation of a prognostic tool to allow patients and clinicians to personalise post-transplant care and alternative approaches to modelling longitudinal transplant function.

Understanding risk associations for graft loss and decline could help early identification of patients for close monitoring, psychosocial support and targeted intervention. Interventions to structure medication-taking are needed, since adherence is worse on the weekend [49]. Among young adults, information technology-based interventions show promise in improving care process self-management outcomes, but their effect on clinical outcomes is less certain [50]. Furthermore, developmentally appropriate interventions will need to be considered for this age-range. Worse outcomes for medication adherence are associated with mental health problems [33]. For UK patients with depression and a chronic physical health problem, National Institute for Health and Clinical Excellence (NICE) recommendations include individual guided self-help through cognitive behavioural therapy [51]. In the low-intensity Improving Access to Psychological Therapies (IAPT) service, a Psychological Wellbeing Practitioner supports patients with frequent but brief coaching [52]. IAPT is effective; depression and anxiety screening scale scores fell with treatment, leading to recovery in ~ 55% of cases. A 5% improvement in employment was observed, associated with lower unemployment benefit costs and higher taxes from employment [53]. Studies evaluating the role of psychological therapies in young adults with transplants are needed.

This is the first UK study to explore risk factors for graft loss as well as eGFR decline for children and young adults. The use of high-quality datasets enabled a long follow-up period, high data completeness and the evaluation of relevant clinic-demographic covariates (excluding immunosuppression regimes). We included second and third grafts in our analyses, which affected 15% of patients. Their inclusion ensures our study population is representative of the child and young adult KRT population in the UK and findings therefore reflect ‘real-world’ data relevant to patients seen in everyday clinical practice. Differing coding systems may limit international comparisons. In our Cox model, there was non-proportionality over time between by kidney disease group, although similar effects were seen in a piecewise model. eGFR decline was modelled over the most recent 5 years of data per patient, assuming linearity. This may not capture the effect of factors associated with initial eGFR decline and may explain why some factors are not common to both analyses, such as HLA mismatch and donor type. Newer alternative techniques, including Bayesian smoothing, allow analysis of all available eGFR data, which may be more representative [54]. We substituted the previous value for missing heights in children, which assumes no growth, thus underestimating the eGFR for that time point. However, the regression line was calculated using all available eGFR data, making this less of an issue. A higher initial eGFR may produce a steeper regression slope and possibly explain these observed associations. The 7-year median follow-up time did not allow analysis of the impact of transition age for different age groups at transplant. We did not conduct a mediation analysis; hence for those variables with potential mediators, total effects only were measured. Our eGFR decline model only explained 5% of the observed variance which may be the result of linear eGFR modelling and/or unmeasured variables. We found an interaction between ethnicity and initial eGFR, where Black ethnicity and better initial function was associated with more rapid rate of eGFR decline. This suggests that this group need careful planning, counselling and clinical support. More work is required to understand these results. We were unable to evaluate residual confounders, such as trends in care such as immunosuppression regimes, surgical advances and kidney allocation policy, although we found no time-period effects in multivariable analyses. We lacked data on cause of graft loss.

## Conclusions

In conclusion, young adulthood is a key risk factor for transplant loss and eGFR decline for UK children and young adults. Risk associations common to graft loss and eGFR decline include female sex, Black ethnicity and glomerular diseases. A higher initial eGFR was associated with less risk of graft loss but faster rate of eGFR decline. It is anticipated that the findings from this study will support health professionals to accurately counsel young people about the life expectancy of their kidney transplant.

## Supplementary Information

**Additional file 1: Supplemental Table 1.** Patient and transplant characteristics with additional data **Supplemental Table 2.** Hazard ratios and 95% confidence intervals for each variable in the graft loss model in univariable analyses, for comparison to the mutually adjusted model shown in Fig. 2. **Supplemental Table 3.** Piecewise multivariable Cox proportional hazards examining associations with death-censored kidney transplant failure in UK patients transplanted under 30 years of age, split at one year of follow-up time. **Supplemental Table 4.** Coefficients and 95% confidence intervals for each variable in the eGFR decline model in univariable analyses, for comparison to the mutually adjusted model shown in Fig. 3.

## Data Availability

The data that support the findings of this study are available from the UK Renal Registry and UK Transplant Registry but restrictions apply to the availability of these data, which were used under license for the current study, and so are not publicly available. Details of the application process for access to individual level (anonymised) UK Renal Registry data for research and non-research purposes are available from https://renal.org/audit-research/how-access-data/ukrr-data. Details of how to access UK Transplant Registry data are available from https://www.odt.nhs.uk/statistics-and-reports/access-data/.

## Abbreviations

CI: Confidence interval
eGFR: Estimated glomerular filtration rate
ESKD: End stage kidney disease
HLA: Human leucocyte antigen
HR: Hazard ratio
IAPT: Improving Access to Psychological Therapies
IQR: Interquartile range
KRT: Kidney replacement therapy
NIHR: National Institute for Health Research
NHS: National Health Service
NHSBT: National Health Service Blood & Transplant
NICE: National Institute for Health and Care Excellence
UKRR: United Kingdom Renal Registry
UKTR: United Kingdom Transplant Registry
