# Supplementary material for: Associations with kidney transplant survival and eGFR decline in children and young adults in the United Kingdom: a retrospective cohort study
Source: BMC Nephrol. 2020 Nov 18;21:492. doi: 10.1186/s12882-020-02156-2 (PMC7672825; doi:10.1186/s12882-020-02156-2)
Supplement: Supplementary file 1 — Additional file 1: Supplemental Table 1. Patient and transplant characteristics with additional data Supplemental Table 2. Hazard ratios and 95% confidence intervals for each variable in the graft loss model in univariable analyses, for comparison to the mutually adjusted model shown in Fig. 2. Supplemental Table 3. Piecewise multivariable Cox proportional hazards examining associations with death-censored kidney transplant failure in UK patients transplanted under 30 years of age, split at one year of follow-up time. Supplemental Table 4. Coefficients and 95% confidence intervals for each variable in the eGFR decline model in univariable analyses, for comparison to the mutually adjusted model shown in Fig. 3. [file 12882_2020_2156_MOESM1_ESM.pdf]

SUPPLEMENTAL MATERIAL

TABLE OF CONTENTS

Supplemental table 1. Patient and transplant characteristics with additional data

Supplemental table 2. Hazard ratios and 95% confidence intervals for each variable in the graft loss model in univariable analyses, for comparison to the mutually adjusted model shown in figure 2.

Supplemental table 3. Piecewise multivariable Cox proportional hazards examining associations with death-censored kidney transplant failure in UK patients transplanted under 30 years of age, split at one year of follow-up time.

Supplemental table 4. Coefficients and 95% confidence intervals for each variable in the eGFR decline model in univariable analyses, for comparison to the mutually adjusted model shown in figure 3.

Supplemental table 1. Patient and transplant characteristics with additional data

Variable	Total n	n	%
**Male sex**	5121	3107	60.7
**Ethnic group**			
White	4918	3955	80.4
Asian		579	11.8
Black		215	4.4
Mixed/other		169	3.4
**Index of multiple deprivation quintile[1]**			
1 (most deprived)	5101	1132	22.2
2		1072	21.0
3		886	17.4
4		992	19.5
5 (least deprived)		1019	20.0
**Primary kidney disease group[2]**			
Glomerular disease	5065	1467	29.0
Systemic diseases affecting the kidney		304	6.0
Familial/hereditary nephropathies		593	11.7
Tubulointerstitial disease		1808	35.7
Miscellaneous kidney disorders		893	17.6
**Late presentation[3]**	3366	867	25.8
**Age when first seen (years) (median, IQR)**	3389	13.1	2.7, 20.0
**Age at KRT start (years) (median, IQR)**	5085	19.1	12.9, 24.1
**Years to KRT start from first nephrology review (median, IQR)**	3370	2.0	0.2. 5.4
**Tertiles of year started KRT (median, IQR)**	5085	2006	2001, 2009
1983-2003	5085	1936	38.1
2004-2008		1607	31.6
2009-2014		1542	30.3
**Start modality**			
Haemodialysis	5102	2011	39.4
Peritoneal dialysis		1839	36.0
Transplant		1252	24.5
**UK Renal Registry database**			
Paediatric database only	5121	300	5.9
Transplanted before transfer to adult services		1662	32.5
Transplanted after transfer to adult services		100	2.0
Adult database only		3059	59.7
**Age at transfer (years) (median, IQR)**	1455	18	17.4, 18.5
**Died**	5121	260	5.1
**Age at death (years) (median, IQR)**	260	27.6	20.3, 32.3
**Transplanted during study period**			
Graft 1	4750	4377	85.5
Graft 2		371	7.2
Graft 3		2	0.04
**Re-transplanted during study period**			
Graft 2	371	369	7.2

Graft 3		2	0.04
**Age at transplant (years) (median, IQR)**			
Graft 1	4377	21.8	14.6, 26.5
Graft 2	740	19.4	14.5, 24.7
Graft 3	4	28.5	24.6, 29.5
**Time from listing to transplant (years) (median, IQR)**	4416	0.8	0.3, 1.8
**Time to transplant from KRT start (years) (median, IQR)**	5085	1.1	0.2, 2.6
None		1113	21.9
<6 months		535	10.5
≥6 months		3435	67.6
**Transplant centre location**			
England	5121	4349	84.9
Scotland		475	9.3
Wales		170	3.3
Northern Ireland		127	2.5
**Year of transplant**			
1998-2003	5121	1291	25.2
2004-2009		1930	37.7
2010-2014		1900	37.1
**Distance from transplant centre (kilometres)[4]**	5093	26.2	10.7, 54.8
**Donor type**			
Live donation	5121	2462	48.1
Donation after brainstem death		2356	46.0
Donation after circulatory death		303	5.9
**Donor age (years) (median, IQR)**	5115	41	27, 49
**Donor Epstein-Barr status**			
Positive	989	375	37.9
Negative		59	6.0
Other/Unknown		555	56.1
**Cytomegalovirus status**			
Donor negative, recipient negative	2123	901	42.4
Donor negative, recipient positive		369	17.4
Donor positive, recipient negative		562	26.5
Donor positive, recipient positive		291	13.7
**Donor cause of death**			
Death from intracranial event	2648	1717	64.8
Death from trauma		642	24.2
Death from infection		110	4.15
Death from other cause		179	6.76
**Donor ethnic group**			
White	4802	4401	91.7
Asian		237	4.9
Black		102	102
Mixed/Other		62	62
**Cold ischaemic time (hours) (median, IQR)**	4651	9.3	2.8, 16.3
**Calculated reaction frequency (%) (median, IQR)[5]**	5118	0	0, 12
**HLA mismatch group[6]**			
0 mismatches	5120	544	10.6

[0 HLA-DR and 0/1 HLA-B] mismatches		2016	39.4
[0 HLA-DR and 2 HLA-B] or [1 HLA-DR and 0/1 HLA-B] mismatches		2272	44.4
[1 HLA-DR and 2 HLA-B] or [2 HLA-DR] mismatches		288	5.6
**First eGFR (mL/min/1.73m^2) (mean, SD)[7]**	4882	62	23
**Median eGFR (mL/min/1.73m^2) (mean, SD)**	4882	58	21
**Rate of eGFR change (mL/min/1.73m^2/year) (mean, SD)**	4487	-3.14	7.69
**Graft failure**	5111	1376	26.9
Follow up time (years) (median, IQR)	5111	7.0	4.0, 10.9
Time to event (years) (median, IQR)	1376	4.2	1.5, 7.6
**Patient death**	4750	238	5.0
Follow up time (years) (median, IQR)	4750	8.8	5.1, 13.0
Time to event (years) (median, IQR)	238	6.6	3.0, 10.6
**Age at event (mean, SD)**	5111	27.5	8.7

IQR – interquartile range; KRT – kidney replacement therapy; SD – standard deviation; HLA – human leucocyte antigen; eGFR – estimated glomerular filtration rate.
Ages are based on dates of birth rounded to mid-month.
[1]Postcodes were used to derive UK–wide index of multiple deprivation quintiles[1].
[2]Primary kidney disease was using a 2012 European coding system[2]. The pediatric diagnosis was used where discordant between pediatric and adult databases[3].
[3]Late presentation defined as ≤90 days from first nephrology review to RRT start.
[4]Distances were created using the Office for National Statistics Postcode Directory[4].
[5]Calculated reaction frequency is defined as the percentage of ABO-identical patients within the donor pool that are HLA incompatible with an individual patient and is dependent on blood group and antibodies[5].
[6]HLA mismatch groups were derived from the UK 2006 National Kidney Allocation scheme[6]. The HLA-A:B:DR mismatches included in each group are as follows:

- [0 HLA-DR and 0/1 HLA-B]
    - 1:0:0, 0:1:0, 1:1:0, 2:0:0, 2:1:0
- [0 HLA-DR and 2 HLA-B] or [1 HLA-DR and 0/1 HLA-B]
    - 0:2:0, 1:2:0, 2:2:0, 0:0:1, 1:0:1, 2:0:1, 0:1:1, 1:1:1, 2:1:1
- [1 HLA-DR and 2 HLA-B] or [2 HLA-DR]
    - 0:2:1, 1:2:1, 2:2:1, 0:0:2, 1:0:2, 2:0:2, 0:1:2, 1:1:2, 2:1:2, 0:2:2, 1:2:2, 2:2:2.

[7]eGFR post-transplant calculated from the first biochemical data recorded by the UK Renal Registry following transplantation. Returns are annual for paediatrics and quarterly for adults.

Supplemental table 2. Hazard ratios and 95% confidence intervals for each variable in the graft loss model in univariable analyses, for comparison to the mutually adjusted model shown in figure 2.

Variable	Hazard Ratio	95% confidence interval		p-value
		Lower	Upper	
**Female sex**	**1.14**	1.03	1.28	0.02
**Live donor (cf. deceased donor)**	**0.79**	0.71	0.88	<0.0001
**Human Leucocyte Antigen mismatches** [One DR & two B locus OR two DR locus mismatches]	**1.46**	1.19	1.79	<0.0001
**Higher first reported eGFR post-transplant** (per 10mL/min/1.73 m^2)	**0.83**	0.80	0.86	<0.0001
**Glomerular diseases**	**1.23**	1.10	1.38	<0.0001
**Age group (cf. 25-29 years)**				
2-4	**0.91**	0.64	1.30	0.6
5-9	**0.54**	0.39	0.74	<0.0001
10-14	**0.87**	0.71	1.06	0.2
15-19	**1.37**	1.18	1.60	<0.0001
20-24	**1.24**	1.07	1.44	0.004
30-34	**0.84**	0.68	1.04	0.1
35-39	**0.82**	0.58	1.16	0.3
40-44	**0.87**	0.43	1.77	0.7
**Ethnicity (compared to White)**				
Asian	**1.04**	0.88	1.22	0.7
Black	**1.33**	1.04	1.71	0.02
Mixed/Other	**0.95**	0.70	1.29	0.7
**Year of transplant (cf. 1998-2005)**				
2006-2010	**0.84**	0.74	0.95	0.005
2011-2014	**0.79**	0.67	0.93	0.005

eGFR – estimated glomerular filtration rate

Supplemental table 3. Piecewise multivariable Cox proportional hazards examining associations with death-censored kidney transplant failure in UK patients transplanted under 30 years of age, split at one year of follow-up time.

Variable	≤1 year				>1 year			
	Hazard Ratio	95% confidence interval Lower	Upper	p-value	Hazard Ratio	95% confidence interval Lower	Upper	p-value
**Female sex**	**0.77**	0.52	1.16	0.2	**0.90**	0.79	1.03	0.1
**Live donor (cf. deceased donor)**	**0.61**	0.39	0.95	0.03	**0.89**	0.78	1.01	0.06
**Human Leucocyte Antigen mismatches** [One DR & two B locus OR two DR locus mismatches]	**1.30**	0.63	2.68	0.5	**1.51**	1.18	1.92	0.001
**Higher first reported eGFR post-transplant** (per 10mL/min/1.73 m^2)	**0.57**	0.49	0.67	<0.0001	**0.86**	0.82	0.89	<0.0001
**Glomerular diseases**	**2.05**	1.34	3.14	0.001	**1.24**	1.08	1.43	0.002
**Age group (cf. 25-29 years)**								
2-4	**1.19**	0.35	4.00	0.8	**0.68**	0.38	1.21	0.2
5-9	**0.62**	0.19	1.97	0.4	**0.49**	0.32	0.74	0.001
10-14	**0.61**	0.24	1.56	0.3	**0.97**	0.76	1.23	0.8
15-19	**1.07**	0.61	1.87	0.8	**1.59**	1.32	1.91	<0.0001
20-24	**1.37**	0.84	2.22	0.2	**1.42**	1.19	1.69	<0.0001
30-34	-	-	-	-	**0.78**	0.62	0.98	0.03
35-39	-	-	-	-	**0.81**	0.57	1.16	0.3
40-44	-	-	-	-	**0.86**	0.41	1.78	0.7
**Ethnicity (compared to White)**								
Asian	**0.90**	0.43	1.86	0.8	**1.03**	0.84	1.26	0.8
Black	**1.15**	0.46	2.85	0.8	**1.52**	1.15	2.01	0.004
Mixed/Other	**1.09**	0.35	3.40	0.9	**1.01**	0.69	1.46	0.98
**Year of transplant (cf. 1998-2005)**								
2006-2010	**1.03**	0.65	1.63	0.9	**1.05**	0.90	1.22	0.5
2011-2014	**0.88**	0.50	1.55	0.7	**1.24**	1.01	1.52	0.04

eGFR – estimated glomerular filtration rate

Supplemental table 4. Coefficients and 95% confidence intervals for each variable in the eGFR decline model in univariable analyses, for comparison to the mutually adjusted model shown in figure 3.

**Variable**	**Coefficient**	**95% confidence interval**		**p-value**
		**Lower**	**Upper**	
**Female sex**	**-1.18**	-1.64	-0.72	<0.0001
**Glomerular diseases**	**-0.72**	-1.23	-0.22	0.005
**First reported eGFR post-transplant (cf. <60 mL/min/1.73m^2)**				
60-90	**-0.78**	-1.25	-0.31	0.001
>90	**-3.88**	-4.65	-3.11	<0.0001
**Age group (cf. 25-29 years)**				
2-4	**-2.78**	-4.48	-1.09	0.001
5-9	**-0.54**	-1.59	0.52	0.3
10-14	**-0.30**	-1.17	0.56	0.5
15-19	**-1.28**	-1.99	-0.58	<0.0001
20-24	**-1.49**	-2.13	-0.86	<0.0001
30-34	**0.81**	0.06	1.55	0.03
35-39	**1.42**	0.25	2.59	0.02
40-44	**2.02**	-0.33	4.37	0.09
**Ethnicity (compared to White)**				
Asian	**0.04**	-0.68	0.77	0.9
Black	**-1.68**	-2.84	-0.53	0.004
Mixed/Other	**0.26**	-1.05	1.57	0.7

eGFR – estimated glomerular filtration rate

REFERENCES

1. Abel GA, Barclay ME, Payne RA. Adjusted indices of multiple deprivation to enable comparisons within and between constituent countries of the UK including an illustration using mortality rates. Bmj Open 2016;6(11)

2. Venkat-Raman G, Tomson CR, Gao Y*, et al.* New primary renal diagnosis codes for the ERA-EDTA. Nephrol Dial Transplant 2012;27(12):4414-4419

3. Hamilton AJ, Casula A, Ben-Shlomo Y*, et al.* The clinical epidemiology of young adults starting renal replacement therapy in the UK: presentation, management and survival using 15 years of UK Renal Registry data. Nephrol Dial Transplant 2018;33(2):356-364

4. Office for National Statistics. *Postcode products*. https://www.ons.gov.uk/methodology/geography/geographicalproducts/postcodeproducts.

5. NHS Blood and Transplant. *Tools and calculators in relation to organ transplantation*. https://www.odt.nhs.uk/transplantation/tools-policies-and-guidance/calculators/.

6. Johnson RJ, Fuggle SV, Mumford L*, et al.* A New UK 2006 National Kidney Allocation Scheme for deceased heart-beating donor kidneys. Transplantation 2010;89(4):387-394
